# Surgery.—Hernia

**Published:** 1905-01-14

**Authors:** 


### SURGERY.—HERNIA.

(*Continued from page* 266.)

**Strangulated Hernia.**—Andrew Clark[5] relates the case of a woman admitted with intestinal obstruction of five days' standing. There was great abdominal distension and fæcal vomiting. A right femoral hernia was present, which she had had for many years; but this was neither tender nor painful, and the patient insisted it had nothing to do with her present illness. The abdomen was filthy and blistered with turpentine stupes; otherwise the cause of obstruction would have been sought by abdominal section, the hernia appearing so unlike a strangulated one. It, however, was cut down on, and a small knuckle of bowel found nipped amongst a mass of omentum. It was in good condition, and was returned after dividing the stricture. The patient died two days later, and the post-mortem showed that the portion of bowel strangulated was the lowest end of the ileum, almost implicating the ileo-cæcal valve. The small bowels were much distended, the large empty. Low[6] relates two cases of Richter's hernia. The first, a woman of 30, was admitted to hospital with intestinal obstruction of three days' standing, and a swelling on the right side in the position of a femoral hernia. On opening this a quantity of foul pus and fæcal matter was evacuated, but no bowel could be found. The abdomen was then opened in the middle line, and a condition of general peritonitis discovered, the peritoneum containing fæcal matter. Near the femoral canal was a coil of intestine about two inches above the ileo cæcal valve, two-thirds of the lumen of which had been nipped. The herniated portion was gangrenous, and had perforated in two places. The bowel was resected and Paul's tubes put in, and the abdominal cavity was irrigated, but the patient died. The second case, that of a woman aged 41, was admitted with symptoms of intestinal obstruction of eight days. The abdomen was flaccid and not tender. There was a swelling in the position of a right femoral hernia. On cutting on to this and opening the sac purulent fluid escaped, but no bowel was present. The abdomen was opened in the middle line, and a condition of general suppurative peritonitis discovered, the pelvis being filled with a purulent fluid similar to that which had escaped from the wound in the groin. A coil of lower ileum was found of which roughly half the circumference had evidently been strangulated, the strangulated portion still retaining the shape in which it had occupied the hernial sac. No obvious perforation was discovered but the herniated portion was covered with thick yellow lymph. The peritoneal cavity was washed out and a drain put in the pelvis and the herniated coil was brought outside the abdomen and fixed with a mesenteric stitch. Four days later this coil looking healthy was replaced. The patient recovered. Burke[7] records the case of a man aged 30 with a right scrotal hernia three days strangulated. On opening the sac a quantity of liquid fæces escaped. The hernia was a large enterocele, the coils being inflamed and matted together, whilst one loop was gangrenous and had perforated. The stricture was divided and the gangrenous bowel—seven inches in length—was resected and the ends joined by immediate suture. The patient recovered. Buchanan[8] relates a case of strangulated umbilical hernia with some unusual symptoms. The patient was a very stout woman ; she had had the hernia for seven years, it had been irreducible one year and strangulated one day. On admission the patient was much collapsed. There was an immense umbilical hernia, very tense and with gangrenous skin covering the greater part of it. Operation was performed without anæsthesia, a vertical incision being made over the whole extent of the hernia, gangrenous intestine and omentum burst into view ; a small incision was made into the bowel and fæcal matter shot out to a distance of several feet, so great was the tension. Most of the matter was expressed from the gangrenous bowel and the opening temporarily closed with a ligature. All the bowel in the sac, a great quantity, was gangrenous. The ring was divided by incision above and below, and an effort made to pull the bowel out but the mesentery would not admit. Gauze was packed round the base of the mass and the patient returned to bed. Vomiting ceased and there were no signs of peritonitis. Two days later numerous incisions were made into the gangrenous bowel, and after about another week the gangrenous mass was cut away, leaving subsequently a deep granulating pit with protruding mucous membrane of bowel above. It was now seen that the parts involved were the large bowel from the cæcum to the splenic flexure, but that the mesocolon had not been long enough to allow the whole bowel to protrude through the ring and the gangrenous process had passed longitudinally through the bowel, leaving the mesocolic part alive and its mucous membrane forming the surface of the protruding mass. The projecting artificial anus was the small bowel at its junction with the cæcum, *i.e.* the ileo-cæcal valve. At a second operation this was freed and implanted end-to-side into the sigmoid flexure and at a third operation the mucous membrane and submucous coats of the large bowel were dissected off leaving about a fourth of the whole to be subsequently dealt with. The patent remained well.

**The Prevention of Ventral Hernia as a Sequel to Abdominal Section.**—Stanmore Bishop[9] mentions the unscientific and somewhat haphazard methods of closing the abdominal wound after section and although fortunately most of the cases are not followed by hernia, still it is sometimes a most distressing sequela to an otherwise successful operation. He points out that the most important structure in the prevention of hernia is the tendon of the three flat muscles of the abdomen forming the sheath of the rectus and linea alba and that when an incision is made in this the contraction of the muscles will cause it to gape but that the rectus muscle will have no effect on it. He further says that it is no use suturing parallel muscular fibres, for they will not unite. The peritoneum must be closed, as if an opening be left for a piece of omentum to get through it may cause a hernia by its tendency to burrow between the superjacent structures. So that in a vertical opening into the abdomen in the middle line or through the rectus the structures that must be sutured separately are the peritoneum for which fine catgut may be used, the aponeurosis and of course the skin. It is no use suturing the muscle or fat. The union of the aponeurosis is the important thing in the prevention of hernia and some method must be adopted which will keep the edges of this structure firmly in apposition against all the straining of the patient until firm union has taken place. This will be from four to six weeks. Any suture material which will remain good for this length of time will be unabsorbable so the sutures must be so placed that when necessary they can be taken out. The following are some of the methods mentioned. The aponeurosis being cleared on its upper surface on one side of the wound and on its under surface on the other side, mattrass sutures of silver wire are put in, brought out through the skin and twisted over a piece of gauze. As the subcutaneous fat wastes from pressure the wire becomes loose, so this is not very satisfactory. Another method, still using silver wire, is to pass it as a figure of eight obliquely through the skin and fat of one side of the wound, and then crossing to the opposite side through the aponeurosis from above downwards, then through the aponeurosis of the same side from below upwards, and finally obliquely through the fat and skin of the opposite side. The wires are twisted over gauze as before, but these do not become loose. In this method the aponeurosis is first freed a little on the under surface on both sides. Milton, of Cairo, unites the aponeurosis with a lock-stitch. The author has found a great difficulty in getting out the secondary thread, there being a tendency for it to break where caught by the primary thread. He uses a modification of it which might be called an interrupted lock-stitch, the primary thread being divided or interrupted, and each pair of ends being brought out through the skin and tied over a piece of gauze. He advises silkworm-gut for this suture, and in removing cuts the interrupted primary threads first and the secondary one then comes out easily. In both of these methods the under surfaces of the aponeurosis are brought into apposition as in the figure of eight suture described above.

**Hernia of the Uterus through the Inguinal Canal.**—Jopson[10] records the case of a woman, aged 27, married, and with three children, who had had a small right inguinal hernia for as long as she could remember, which always disappeared on lying down. A week before admission to hospital, and whilst she was washing, a much larger protrusion than usual made its appearance, accompanied with severe pain. There was neither vomiting nor constipation. On admission she was in excellent condition and there was in the right inguino-labial region a hard, irreducible, somewhat tender hernia the size of half a fist. There being no intestinal symptoms it was thought to be an epiplocele. An operation was performed, and on opening the sac the appearances were somewhat confusing, and in endeavouring to unfold what was still thought to be omentum, it split longitudinally, about three-quarters of an ounce of yellow odourless pus escaping. After this was sponged away an ovary was seen projecting to the right side of the neck of the mass, and what had been supposed to be large intestine flattened out was the broad ligament and Fallopian tube. The herniated mass was then seen to be the uterus turned over forward, the supra-vaginal portion running backward, downward, and inward towards the cervix. Sutures being applied to broad ligaments, tubes, and supra-vaginal portion of the uterus, the parts were cut away. The infected stump of the uterus was sutured to the external abdominal ring. The patient made a good recovery. The author concludes his paper with a brief review of the literature cf the subject.

[5] Brit. Med. Jour., May 7, 1904. [6] Lancet, Sept. 24, 1904. [7] Brit. Med. Jour., April 16, 1904. [8] Med. Rec., Oct. 8, 1904. [9] Brit. Med. Jour., July 9, 1904. [10] Annals of Surg., July 1904.

